# A Systematic Review of the Methods of Assessment of Gastro-Oesophageal Reflux in Anaesthetized Dogs

**DOI:** 10.3390/ani11030852

**Published:** 2021-03-18

**Authors:** Anna Carolina Fernandez Alasia, Olivier Levionnois, Mathieu Raillard

**Affiliations:** 1 School of Veterinary Science, Faculty of Science, Evelyn Williams Building No B10, The University of Sydney, Sydney, NSW 2006, Australia; afer9691@uni.sydney.edu.au; 2 Anaesthesiology Section, Department of Clinical Veterinary Sciences, Vetsuisse Faculty, University of Berne, 3012 Berne, Switzerland; olivier.levionnois@vetsuisse.unibe.ch

**Keywords:** anaesthesia, complications, dogs, gastro-oesophageal reflux, regurgitation, risk

## Abstract

**Simple Summary:**

Regurgitation and gastro-oesophageal reflux (GOR) are common complications in dogs under anaesthesia. We reviewed the definitions and methods of GOR assessment in anaesthetized dogs published in 22 scientific papers to assess if studies were comparable (i.e., looking at the same thing). The definition of GOR implied the presence of fluids not reaching the mouth or nose in the oesophagus in all studies. Most studies measured the acidity in the oesophagus to state if fluids were present or not. The probes were not always placed in the same location and definitions varied. This means that it is complicated to compare findings of the different studies.

**Abstract:**

We reviewed the definitions and methods of assessment of gastro-oesophageal reflux (GOR) in anaesthetized dogs. Three databases were used. Titles and abstracts were screened by two of the authors independently. A total of 22 studies was included in the analysis. The definition of GOR implied the presence of fluids not reaching the mouth or nose in the oesophagus in all studies. Most studies considered a change in pH using oesophageal pH meters as the sole method of assessment. Calibration of the pH probe was inconsistently reported. The position of the tip of the oesophageal probe was inconsistent and not always precisely described. The correct positioning in the intended location was verified in a limited number of studies. Some studies considered that GOR had happened for changes in pH below 4.0 or above 7.5 while others considered that GOR had happened when the pH dropped below 4.0 only. Some studies stated that the pH change had to be sustained for a minimum period of time (20 or 30 s) whereas others did not mention any duration. The variability of definitions and methods of assessment of GOR in anaesthetized dogs precludes meaningful comparison of the findings. Re-evaluation and uniformization of the methods appear necessary.

## 1. Introduction

Regurgitation and gastro-oesophageal reflux (GOR) are common complications in dogs undergoing general anaesthesia and can lead to significant morbidity and mortality. The literature highlights several risk factors (i.e., age, body weight, and type of surgery) and reports that the incidence of regurgitation and GOR may be influenced by a number of interventions (i.e., pre-operative fasting, positioning, and drugs). Reported incidences seem to vary reasonably for regurgitation [from 0.96% [1] to 5.5% [2],] but enormously for GOR [from 5% [3] to 87.5% [4]]. Such a huge variability is rather surprising. Before contrasting published findings and interventions aimed to reduce the development of regurgitation and GOR, it seems legitimate to question the methods used in the scientific literature.

The aim of this study was to review the definitions and methods of assessment of GOR in anaesthetized dogs in clinical veterinary practice.

## 2. Materials and Methods

The PRISMA (Preferred Reporting Items for Systematic Reviews and Meta-Analyses) checklist was used.

The review protocol was not registered. The search was electronic. The search strategy was as follows: ((dogs OR canines OR dog OR canine) AND (anaesthetized OR anesthetized OR anaesthetize OR anesthetize OR anaesthetise OR anaesthesia OR anesthesia) AND (gastro-oesophageal reflux OR GER OR GOR OR gastroesophageal reflux or gastro-oesophageal reflux)). Three databases were used: Pubmed, Embase, and Scopus. The search was last performed on the 6 December 2020. References of relevant publications were also consulted. Titles and abstracts were screened by two of the authors independently. There was no *a priori* year restriction. Case reports, case series, conference papers. and non-English publications were excluded. The focus of this review being anaesthesia in clinical veterinary practice, studies including dogs as an experimental model and studies not focusing on GOR during the intra-anaesthetic period were excluded.

Information extracted included: (1) definitions and endpoints used in the article for regurgitation; (2) definitions and endpoints used in the article for GOR; (3) methods of assessment of regurgitation and GOR; (4) pH probe calibration if appropriate (method and timing); (5) equipment positioning, time of insertion, and removal; (6) verification of the appropriate location of the probe and timing of the check; (7) frequency of measurements; and (8) particular precautions (to limit probe dislodgement or effort to limit the iatrogenic GOR and regurgitation).

Descriptive statistics were performed where appropriate.

## 3. Results

**Figure 1:**
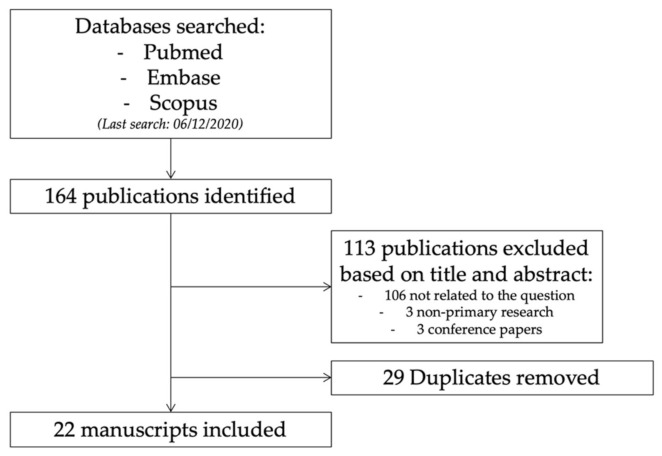
Consort diagram for search strategy.

The consort diagram for the search strategy is presented in Figure 1. A total of 164 studies were assessed for eligibility: 76 in Pubmed (56 were excluded: 54 not related to the question, one non-primary research, one abstract), 69 in Embase (48 were excluded: 43 not related to the question, two non-primary research, three abstracts); 19 in SCOPUS (9 were excluded as not related to question). Duplicates were removed. A total of 22 submissions was reviewed. 

The 22 manuscripts assessed in this review are reported in Table 1. A total of 10/22 studies did not talk about regurgitation. The definition of regurgitation generally implied the passive and visible discharge of fluid from the mouth or nose. However, one study considered a change in pH in the pharynx as an episode of regurgitation. In that study, pharyngeal pH was measured when GOR episodes were identified, to evaluate the spread of the reflux.

The definition of GOR implied the presence of fluids not reaching the mouth or nose in the oesophagus in all studies. The portion of the oesophagus considered was infrequently reported in the definition: “distal” or “caudal” or “lower” oesophagus in 8/22 papers. Most studies (20/22) identified the development of GOR through a change in oesophageal pH. This was the sole method of assessment in 18/22 studies, while two studies used oesophagoscopy on top of the pH meter to visualise if reflux was present (i.e., non-acid reflux not causing pH changes). One study used a combined pH/impedance probe and considered a 50% decrement in Ohms seen in two consecutive impedance channels in the distal oesophagus for >2 s compared with the pre-episodic oesophageal baseline recording. One study retrospectively assessed the presence of gas, fluid, or alimentary content on CT (computed tomography) images.

In the 20 studies considering pH changes, 14 considered that GOR had happened for changes in pH “below 4.0 or above 7.5” while six considered that GOR had happened when the pH dropped below 4.0 only. The study using impedance changes categorized the pH of the reflux but this was not the criteria used to state if GOR had happened. In addition, for GOR to be confirmed, six studies stated that the pH change had to be sustained for a minimum period of time (20 or 30 s) whereas the others did not mention any duration in pH change.

Irrespective of definitions and main outcome measures, 21/22 of the studies used pH meters. Calibration was reported in only 14 studies. Two-point calibration (generally with buffers of pH 1 or 4 and pH 7) was used.

The position of the tip of the oesophageal probe was inconsistent and not always described with precision. The distance between the lower incisors and the cranial margin of the 10th rib was externally measured with the dogs in lateral recumbency in 14 studies. However, the probe was advanced that distance in only 10/14 studies, whereas it was advanced that distance minus 5 cm in 4/14. The distance between the upper canine and the distal border of the ninth rib (in a straight line with the dogs’ head and neck in a “normal position”) was measured in two studies where the probe was advanced that distance. It was estimated to position the tip of the probe about 7 cm cranially to the lower oesophageal sphincter in a “preliminary trial” where radiography was used. The probe was advanced until the ninth rib in one study. It was retrieved from the stomach, 6 cm cranially to the lower oesophageal sphincter (visualised through video-oesophagoscopy) in one study. The pH was measured at three different levels in the oesophagus (thoracic inlet, fifth and ninth rib) in one study. The location of the probe placement was not reported in 2/21 studies. The correct positioning of the probe in the intended location was verified through endoscopy, chest radiographs, or fluoroscopy in only 5/21 studies. The permanence of the probe in the appropriate location at the beginning and at the end or throughout the study was never checked.

The full results are presented in Appendix A.

## 4. Discussion

A variety of definitions and methods of assessment of GOR in anaesthetized dogs is present in the literature. Although oesophageal pH measurement is the method most commonly used, calibration, position of the probe, and cut-off values of pH to differentiate between GOR or not are inconsistent.

The method of analysis of pH is relative. This means that pH meters need to be appropriately calibrated [24]. The pH calibration curve is a combination of two curves, the pH and the pOH curves and is not linear. A slight deviation in the range of pH 6–8 is expected [24]. Generally, a pH meter should be calibrated every 2 to 3 h using at least two buffer solutions with known pH values close to the expected pH to be measured [24]. When considering a wide range of pH, two-point calibration is not sufficient [24]. Also, given the effects of temperature on pH measurements [24], the question of calibrating the instruments at body temperature exists. In the present review, several studies used oesophageal pH meters without reporting any calibration. Studies reporting calibration used a two-point calibration, mostly using buffers of 1 or 4 and 7 while eight studies were defining GOR as a pH change below 4 or above 7.5. The accuracy of relevant pH values presented is questionable. Each pH unit change represents a 10-fold change in the hydrogen or hydroxyl ions concentration. The calibration might be less important if definitions considered sudden changes in pH as a proxy biomarker of GOR instead of numerical cut-off values.

Anatomic landmarks were commonly used to estimate the position of the lower oesophageal sphincter and the length of the probe to advance in the oesophagus. Most studies used the description proposed by Waterman and Hashim [25]. However, the length of the oesophageal probes advanced was variable. Also, the position of the tip of the probe was rarely checked. Depending on the volume of the refluxate, actual GOR could be missed, for example, in the case of pH measurements in the proximal oesophagus if material is present only caudally or if the tip of the probe is not in the liquid phase in the dependent oesophagus. Furthermore, there was no consensus on the definition of GOR and pH cut-off values of the refluxate. Although acid reflux seems to be observed more frequently than alkaline reflux in the articles examined, actual incidence of GOR might be underestimated in some studies. These points make comparisons between studies challenging from the perspective of actual incidence or efficacy of measures taken.

Consequences of GOR can include oesophagitis, oesophageal stricture, and aspiration pneumonia [26,27]. Evidence in humans suggests that mixed reflux (acid mixed with bile acids) is more harmful to the oesophageal mucosa than acid reflux alone [28] and that bile reflux in the oesophagus can occur over a wide range of pH (2–8) [29]. Although the actual importance of this information in dogs undergoing a single anaesthesia and GOR episode is not known, the clinical relevance of changes in oesophageal pH as a sole marker of GOR is questionable. The position of the tip of the probe as well as the cut-off values of pH used to assess the presence vs absence of GOR might require re-evaluation. Although pH-metry seems commonly used, likely because of its wide availability and low cost, this technique alone might be insufficient. Combined pH/impedance probes with multiple impedance channels to evaluate the spread of the refluxate in the oesophagus, similar to the product used by Zacuto et al. (2012) [16] or Tarvin et al. (2016) [30], calibrated and in a well identified (checked) location, associated with biological analysis of the refluxate might offer a more relevant picture of GOR in anaesthetized dogs.

## 5. Conclusions

The variability of the GOR incidence found in the literature is likely due to a variety of factors (i.e., anaesthetic depth, transport, and position). However, the multiple definitions and methods of assessment of GOR in anaesthetized dogs present in the literature preclude meaningful comparison of the findings. Re-evaluation and uniformization of the methods seem necessary. Some aspects might warrant further investigation: (1) the relevance of the volume of the material regurgitated and how long the reflux remains in the oesophagus for; (2) the impact of anaesthesia on oesophageal motility; and (3) the importance of anaesthetic depth and the presence of monitoring equipment in the oesophageal lumen on the incidence of GOR.

## Figures and Tables

**Table 1:** Reference number, authors, year of publication, and journal of the 22 manuscripts included in the review of the methods of assessment of gastro-oesophageal reflux (GOR) in anaesthetized dogs.

Ref.	Authors	Year	Journal
[5]	Roush JK, Keene BW, Eicker SW et al.	1990	*Vet. Surg.*
[6]	Galatos AD, Raptopoulos D	1995	*Vet. Rec.*
[7]	Galatos AD, Raptopoulos D	1995	*Vet. Rec.*
[2]	Wilson DV, Evans AT, Miller R	2005	*Am. J. Vet. Res.*
[8]	Wilson DV, Boruta DT, Evans AT	2006	*Am. J. Vet. Res.*
[9]	Wilson DV, Evans AT, Mauer WA	2006	*Am. J. Vet. Res.*
[10]	Wilson DV, Tom Evans A, Mauer WA	2007	*Vet. Anaesth. Analg.*
[11]	Wilson DV, Evans AT	2007	*Vet. Anaesth. Analg.*
[12]	Anagnostou TL, Savvas I, Kazakos GM et al.	2009	*Vet. Anaesth. Analg.*
[13]	Panti A, Bennett RC, Corletto F et al.	2009	*J. Small Anim. Pract.*
[14]	Favarato ES, de Souza MV, dos Santos Costa PR et al.	2011	*Vet. Res. Commun.*
[15]	Favarato ES, Souza MV, Costa PR et al.	2012	*Res. Vet. Sci.*
[16]	Zacuto AC, Marks SL, Osborn J et al.	2012	*J. Vet. Intern. Med.*
[17]	Johnson RA	2014	*Vet. Anaesth. Analg.*
[18]	Anagnostou TL, Savvas I, Kazakos GM et al.	2015	*Vet. Anaesth. Analg.*
[3]	Savvas I, Raptopoulos D, Rallis T	2016	*J. Am. Anim. Hosp. Assoc.*
[19]	Anagnostou TL, Kazakos GM, Savvas I et al.	2017	*Vet. Anaesth. Analg.*
[20]	Shaver SL, Barbur LA, Jimenez DA et al.	2017	*J. Am. Anim. Hosp. Assoc.*
[21]	Torrente C, Vigueras I, Manzanilla EG et al.	2017	*J. Vet. Emerg. Crit. Care*
[22]	Viskjer S, Sjostrom L	2017	*Am. J. Vet. Res.*
[4]	Lambertini C, Pietra M, Galiazzo G et al.	2020	*Vet. Sci.*
[23]	Benzimra C, Cerasoli I, Rault D et al.	2020	*J. Vet. Sci.*

## Data Availability

Data is contained within the article.

## Appendix A

**Table A1:** Definitions and endpoints of regurgitation and gastro-oesophageal reflux (GOR), methods of assessment of GOR +/− regurgitation, pH probe calibration (method and timing), equipment positioning and placement check, frequency of measurements, and particular precautions in 22 scientific publications evaluating aspects of GOR in anaesthetized dogs.

Article	Gastro-Oesophageal Regurgitation Definition and Endpoint(s)	Gastro-Oesophageal Reflux Definition and Endpoint(s)	Methods of Assessment of GOR +/− Regurgitation	pH Probe Calibration 1. Method 2. Timing	1. Equipment Positioning 2. Time Insertion 3. Time Removal	1. Placement Check 2. Timing	Frequency of Measurement	Particular Precautions
[5] Roush et al., 1990	N/A	“Reflux of gastric contents and bile salts into the oesophageal lumen” Oesophageal pH < 4.0 or >7.5	Oesophageal pH meter	1. Not reported 2. N/A	1. Oesophageal pH measurements made at the thoracic inlet, heart base (level of the fifth rib), and gastroesophageal sphincter (level of the tenth rib) 2. N/A 3. N/A	1. Not reported 2. N/A	Measurements at minutes 10 and 30, and every 30 min thereafter until recovery from anaesthesia	Not reported
[6] Galatos and Raptopoulos 1995	Not defined but reported in two dogs	Lower oesophageal pH < 4.0 or >7.5	Oesophageal pH meter	1. Not reported 2. N/A	1. Probe introduced in the oesophagus via the oropharynx the distance measured from upper canine and distal border of ninth rib in a straight line with head and neck in a “normal position” (estimated about 7 cm cranial to lower oesophageal sphincter in a “preliminary trial” where radiography was used) 2. Within 5 min from anaesthesia induction 3. After the end of oesophageal pH monitoring, electrode advanced in the stomach for gastric pH measurement	1. Not reported 2. N/A	Monitored continuously, recorded every 5 min from at least 60 min (or until the completion of the procedure) after induction of anaesthesia	Not reported
[7] Galatos and Raptopoulos 1995	Not defined but reported in one dog	Lower oesophageal pH < 4.0 or >7.5	Oesophageal pH meter	1. Not reported 2. N/A	1. Probe introduced in the oesophagus via the oropharynx the distance measured from upper canine and distal border of ninth rib in a straight line with head and neck in a “normal position” (estimated about 7 cm cranial to lower oesophageal sphincter in a “preliminary trial” where radiography was used) 2. Within 5 min from anaesthesia induction 3. After the end of oesophageal pH monitoring, electrode advanced in the stomach for gastric pH measurement	1. Not reported 2. N/A	Monitored continuously, recorded every 5 min from at least 60 to 140 min after induction of anaesthesia	Not reported
[2] Wilson et al., 2005	“When refluxed fluid is of sufficient volume to reach the pharynx and even drain from the mouth. (…) Passive discharge of liquid from the mouth or nose of a dog during anaesthesia” Direct visualization and pH measurement	“Reflux of gastric contents into the oesophagus” Oesophageal pH decreases to <4.0 (acidic reflux) or increases to >7.5 (biliary reflux)	Oesophageal pH meter +/− measurement pH of fluids discharged	1. Not reported 2. N/A	1. Oesophageal probe taped to an oesophageal stethoscope advanced through the oropharynx and into the oesophagus to the distance between the incisor tooth (on the lower hemimandible) and cranial margin of the head of the 10th rib across the angle of the mandible measured externally 2. After induction of anaesthesia at a time when the dog was judged to be at a sufficiently deep plane of anaesthesia to tolerate insertion 3. Removed prior to extubation	1. Not reported 2. N/A	Continual collection of data for the duration of anaesthesia	Not reported
[8] Wilson et al., 2006	“Passive discharge of liquid from the mouth or nose” Direct visualization and pH of any fluid that dripped from the mouth or nose measured	Decrease in oesophageal pH to <4 (reflux of gastric acid) or an increase to >7.5 (reflux of bile) for a period of ≥30 s	Oesophageal pH meter	1. Two-point calibration (buffer solutions pH 1 and 7) 2. Within 2 h prior to use	1. Tip of the probe taped to an oesophageal stethoscope and advanced through the oropharynx into the oesophagus the distance between the incisor tooth on the lower jaw and the cranial margin of the 10th rib (measured externally) 2. After induction of anaesthesia and endotracheal intubation 3. Prior to extubation	1. Not reported 2. N/A	Continuous monitoring	Probe placement was performed by 1 of 3 trained people The probe was affixed in place
[9] Wilson et al., 2006	“Passive discharge of liquid from the mouth or nose” Direct visualization and pH of any fluid that dripped from the mouth or nose measured	Decrease in oesophageal pH to <4 (reflux of gastric acid) or an increase to >7.5 (reflux of bile) for a period of ≥30 s	Oesophageal pH meter	1. Two-point calibration (buffer solutions pH 1 and 7) 2. Within 2 h prior to use	1. Tip of the probe taped to an oesophageal stethoscope and advanced through the oropharynx into the oesophagus the distance between the incisor tooth on the lower jaw and the cranial margin of the 10th rib (measured externally) 2. After induction of anaesthesia and endotracheal intubation 3. Prior to extubation	1. Not reported 2. N/A	Continuous monitoring	Probe placement was performed by 1 of 3 trained people The probe was affixed in place
[10] Wilson et al., 2007	“Passive discharge of liquid from the mouth or nose” Direct visualization and pH of any fluid that dripped from the mouth or nose measured	Decrease in oesophageal pH to <4 (reflux of gastric acid) or an increase to >7.5 (reflux of bile) for a period of ≥30 s	Oesophageal pH meter	1. Two-point calibration (buffer solutions pH 1 and 7) 2. Within 2 h prior to use	1. Tip of the probe taped to an oesophageal stethoscope and advanced through the oropharynx into the oesophagus the distance between the incisor tooth on the lower jaw and the cranial margin of the 10th rib (measured externally) 2. After induction of anaesthesia and endotracheal intubation 3. Prior to extubation	1. Not reported 2. N/A	Continuous monitoring	Probe placement was performed by 1 of 3 trained people The probe was affixed in place
[11] Wilson and Evans 2007	“Passive discharge of liquid from the mouth or nose of a dog during general anaesthesia” Direct visualization	Observation of 30 s or longer of a decrease in oesophageal pH to <4 (reflux of gastric acid)	Oesophageal pH meter	1. Two-point calibration (buffer solutions pH 1 and 7) 2. Within 2 h prior to use	1. Tip of the probe advanced through the oropharynx into the oesophagus the pre-measured distance between the incisor tooth on the lower jaw and the cranial margin of the head of the 10th rib measured externally 2. Probe inserted after induction of anesthesia and endotracheal intubation 3. Probe removed prior to extubation	1. Not reported 2. N/A	Continual data collection	Probe placement performed by one of three trained people. The probe was affixed in place.
[12] Anagnostou et al., 2009	N/A	pH values of >7.5 (alkaline reflux) or <4 (acid reflux) in the lower oesophagus	Oesophageal pH meter	1. Two-point calibration (buffer solutions pH 4 and 7) 2. “Previously”	1. pH-meter inserted into the oesophagus through the oral cavity. Length determined subtracting 5 cm from pre-measured distance between lower incisor tooth (animal in left lateral recumbency) and anterior border of the head of the 10th rib through the angle of the mandible 2. Immediately after intubation of the trachea and connection of the endotracheal tube to the anaesthetic machine 3. After completion of 1 h of continuous oesophageal pH monitoring	1. Not reported 2. N/A	Monitored continuously for 60 min after induction of anaesthesia	Dogs placed in dorsal recumbency immediately after securing the probe. During procedures and position changes, special attention was paid to avoiding application of pressure to the abdominal wall
[13] Panti et al., 2009	“Return of partially digested food from the stomach to the mouth”	Abrupt decrease in distal oesophageal pH below 4	Oesophageal pH meter	1. Two-point calibration (buffer solutions pH 4 and 7) 2. Approximately every three cases and at least once a week	Probe placed inside a protective polythene tube. and inserted into the distal oesophagus. The probe was inserted into the oesophagus, with the tip at the level of the ninth rib, which is about 7 cm rostral to the LOS (Position of the lower oesophageal sphincter estimated the length between the incisor of the lower jaw and the cranial border of the head of the 10th rib, measured externally with the animal in lateral recumbency) 2. Immediately after induction of anaesthesia 3. Not reported	1. Not reported 2. N/A	Recorded every five minutes during anaesthesia	The same operator positioned the oesophageal pH probe in each dog
[14] Favarato et al., 2011	N/A	“Presence of acid reflux in the oesophagus” Oesophageal pH < 4 and visualisation of content through video-oesophagoscopy	Oesophageal pH meter and video-oesophago-scopy	1. Two-point calibration (buffer solutions pH 1.0 and 7.0) 2. Maximum 1 h before the procedure	1. Close and cranially to the oesophago-gastric juntion (measuring the distance between the mandible incisor teeth and the cranial border of the tenth rib through the angle of the mandible after the pre-anaesthetic medication, the animals positioned in left lateral recumbency) 2. “Intra-operatively” 3. Catheter removed immediately after esophagoscopy	1. Video-oesophago-scopy 2. Immediately after surgery	Constantly monitored, variations recorded	Dogs maintained in a dorsal horizontal recumbency, on a surgical table, during the surgical procedure. Lateral decubitus after the end of surgery. No position changes allowed during the evaluation period
[15] Favarato et al., 2012	N/A	pH lower than 4 considered an acid reflux episode; confirmation of the non-acid reflux obtained by esophagoscopy conducted on all the animals immediately after surgery to evaluate the presence of visible reflux in the oesophageal lumen	Oesophageal pH meter and video-oesophago-scopy	1. Not reported 2. N/A	1. “Close and cranially to the oesophago-gastric junction” 2. N/A 3. N/A	1. Not reported 2. N/A	Monitoring throughout the anaesthetic procedure, with all the pH variations recorded	N/A
[16] Zacuto et al., 2012	N/A	50% decrement in ohms seen in 2 consecutive impedance channels in the distal oesophagus for >2 s from the pre-episodic oesophageal baseline recording. The pH of the refluxate was classified as strongly acidic (pH < 4.0), weakly acidic (4.0 < pH < 7.0), or nonacidic (pH ≥ 7.0)	Oesophageal multi-use pH/impedance probe	1. Probe calibrated in buffer solutions of pH 4.0 and 7.0 2. Within 10 min of use	1. The esophageal probe was introduced into the esophagus via the oral cavity by use of a loop snare passed through the biopsy channel of a fibreoptic endoscope The probe was advanced into the greater curvature region of the stomach to record gastric pH for 2 min before probe placement in the distal esophagus. After recording of gastric pH, the pH sensor on the esophageal probe was positioned 6 cm proximal to the gastroesophageal junction in all dogs, and no portion of the probe traversed the LES during the recording period 2. Immediately after induction 3. Immediately before extubation	1. Oesophago-scopy 2. During placement	Esophageal pH and impedance were recorded throughout the surgical procedure	Probe placement was performed in all dogs by 1 of 2 investigators skilled in endoscope handling to ensure consistency in the positioning of the probe The probe then was secured in place using tape wrapped around the maxilla
[17] Johnson 2014	N/A	≥30 s decrease in oesophageal pH to <4 (gastric acid reflux) or an increase to >7.5 (bile reflux)	Oesophageal pH meter	1. Two-point calibration (buffer solutions pH 4 and 7) 2. Within 1 h before use	1. The distance between the mandibular incisor tooth and cranial margin of the 10th rib was measured externally. The probe tip was advanced this distance through the oropharynx into the oesophagus 2. Following induction of anaesthesia 3. Removed before tracheal extubation	1. Not reported 2. N/A	Continual data collection	The probe was taped to the endotracheal tube and just behind the maxillary canines
[18] Anagnostou et al., 2015	N/A	Lower oesophageal pH values of >7.5 (alkaline reflux) or <4 (acid reflux)	Oesophageal pH meter	1. Two-point calibration (buffer solutions pH 4 and 7) 2. “previously”	1. Probe inserted into the oesophagus through the oral cavity; length subtracting 5 cm from the pre-measured distance between the lower incisor tooth and the anterior border of the head of the 10th rib through the angle of the mandible (animal in left lateral recumbency) 2. Immediately after intubation of the trachea and connection of the endotracheal tube to the anaesthetic machine 3. Oesophageal probe removed on completion of surgery before discontinuing administration	1. Not reported 2. N/A	Monitored continuously after induction of anaesthesia and throughout surgery	Immediately after securing the probe in place, the animal was placed in dorsal recumbency. During introduction of the pH-measuring probe, change of the animals’ position, and aseptic preparation (clipping, scrubbing), special attention was paid to avoiding application of excessive pressure to the abdominal wall
[3] Savvas et al., 2016	pH-change at the pharynx, measured when GOR was observed	Oesophageal pH < 4 or >7.5	Oesophageal +/− upper oesophageal pH meter	1. Not reported 2. Not reported	1. Oesophageal probe: 5 cm above the lower oesophageal sphincter, estimated by measuring the length from lower jaw incisor tooth to the anterior border of the head of the tenth rib 2. Following intubation of the trachea 3. pH recording was discontinued just prior to extubation NB: Second pH probe with its tip at the upper oesophageal sphincter (at the level of the larynx) when GOR observed	1. Not reported 2. N/A	Constantly monitored, recorded every 5 min	No transportation of the animals to another operation room. All possible precautions were taken to prevent increases in intra-abdominal pressure (from manipulations of the animals during handling and surgery)
[19] Anagnostou et al., 2017	“Reflux material observed at the external nares or in the mouth”	Whenever a pH value > 7.5 (alkaline reflux) or <4.0 (acid reflux) was recorded	Oesophageal pH meter	1. Two-point calibration (buffer solutions pH 4 and 7) 2. Before each use	1. Probe introduced into the oesophageal lumen through the oral cavity. The distance between the lower incisor teeth (animal in left lateral recumbency) and the anterior border of the head of the 10th rib through the angle of the mandible was measured considered to correspond to the approximate location of the posterior oesophageal sphincter; the final length of the pH measuring probe that was inserted into the oesophageal lumen was calculated by subtracting 5 cm from this measured distance 2. Immediately after tracheal intubation and connection of the endotracheal tube to the anaesthetic machine 3. After completion of surgery, administration of halothane discontinued and pH probe withdrawn	1. Not reported 2. N/A	Continuous monitoring	Immediately after securing the probe in place, the animal was placed in sternal recumbency on a horizontal table. Application of excessive pressure to the abdominal wall or to the surgical area that could potentially cause GOR was avoided at all times and especially during introduction of the pH measuring probe, change of recumbency, clipping and scrubbing.
[20] Shaver et al., 2017	“Gastric contents refluxing to the oropharynx”	Prolonged (>20 s) decreases (<4.0) or increases (>7.5) in oesophageal pH	Oesophageal pH meter	1. Two-point calibration (buffer solutions pH 4 and 7) 2. Immediately before use	1. Probe inserted orally and advanced a distance measured from the incisors to the cranial margin of the tenth rib to result in a predictable location just proximal to the lower oesophageal sphincter 2. After induction of anaesthesia 3. Just prior to patient endotracheal extubation	1. Thoracic radiograph 2. Immediately after placement	Continuous monitoring of oesophageal pH, recorded at 5 min intervals	Probe placed by a single surgeon Medical tape was fixed to the probe at the level of the first premolar and stapled to the dog’s upper lip using surgical staples
[21] Torrente et al., 2017	“Passive ejection of gastric or oesophageal content from the mouth or nose” Direct visualisation	Oesophageal pH < 4 was considered an acid reflux event	Oesophageal pH meter	1. Not reported 2. N/A	1. Oesophageal probe during anaesthesia and just before return to full consciousness (no more detail) 2. N/A 3. N/A	1. Not reported 2. N/A	Not reported	Not reported
[22] Viskjer and Sjostrom 2017	“Any visible regurgitation of gastric content through the mouth” Direct visualisation	“Reflux of gastric content into the oesophagus” pH value < 4.0 in the distal oesophagus	Oesophageal pH meter	1. Two-point calibration (buffer solutions pH 1.07 and 7.01) 2. Before probe placement in each dog	1. pH catheter introduced through the oropharynx into the oesophagus, advanced a fixed distance into the oesophagus on the basis of the distance from the most rostral incisor tooth on the mandible to the cranial margin of the head of the 10th rib 2. N/A 3. After the surgical procedure	1. The position of the tip of the catheter in the distal portion of the oesophagus was confirmed radiographically 2. N/A	Measurement every fourth second (frequency 0.25 Hz)	Catheter secured to the mandible with adhesive tape. Dogs’ position and duration of anaesthesia recorded
[4] Lambertini et al., 2020	N/A	“Reflux of gastric content into the oesophagus” Decrease in oesophageal pH to a value <4.0 (acidic reflux) or as an increase to a value >7.5 (biliary reflux) for at least 30 s	Oesophageal pH meter	1. Two-point calibration (buffer solutions pH 4.0 and 7.0) 2. 1 h prior to the procedure	1. Probe into the oesophagus for a length equal to the distance between the incisor tooth and the cranial border of the 10th rib 2. Soon after intubation 3. Probe removed at the end of the endoscopy of the upper gastrointestinal tract in the END group or immediately before extubation in the ORT group	Confirmed directly under endoscopic view in the END group dogs, or by fluorospic evaluation (lateral view of the thorax) in the ORT group dogs. Fluoroscopic examination carried out, passing through the surgical table from below, before the preparation of the surgical field	Continuously recorded every 1 s from the probe insertion up to its removal	Probe fixed to the canine tooth with tape in order to prevent its dislodgement. Dogs in END group maintained in left lateral recumbency. Dogs in the ORT group into the recumbency required for the surgical procedure. (recorded). Surgical table always parallel to floor.
[23] Benzimra et al., 2020	N/A	Presence gas, fluid, or alimentary contents in the caudal oesophagus on CT images	Oesophageal content retrospectively assessed on plain and myelo-CT scans	N/A	CT examination of the thoracolumbar spine in which the field of view allowed visualization of the full thoracic path of the oesophagus and the entire stomach. Caudal portion of the thoracic oesophagus evaluated from the base of the heart to the cardia	N/A	Retrospective analysis	All qualitative assessments were performed by a third-year ECVDI resident using a dedicated DICOM viewer
